# Prevalence and associated factors of glomerular hyperfiltration among adult stable sickle cells in Kinshasa, DR Congo

**DOI:** 10.1080/0886022X.2024.2407888

**Published:** 2024-09-27

**Authors:** Yannick Engole Mompango, Justine Bukabau Busanga, Jean Robert Makulo Rissassy, Yannick Nlandu Mayamba, Brady Makanzu, Aliocha Nkodila, Tresor Tshiswaka, Vieux Mokoli Momeme, Augustin Longo Luzayadio, Marie France Mboliasa Ingole, François Kajingulu Musungayi, Shekinah Fwana, Cedric Ilunga Kabemba, Clarisse Nkondi Nsenga, Chantal Zinga Vuvu, Nazaire Nseka Mangani, Ernest Sumaili Kiswaya

**Affiliations:** a Nephrology Unit, Kinshasa University Hospital, Kinshasa, XI, Democratic Republic of the Congo; b Specialized Clinics in Kinshasa, Kinshasa, Democratic Republic of the Congo; c Cardiology Unit, Kinshasa University Hospital, Kinshasa, XI, Democratic Republic of the Congo

**Keywords:** Hyperfiltration, sickle cell disease, albuminuria, nephropathy, CKD-EPI, cystatine C

## Abstract

**Introduction:**

Glomerular hyperfiltration is highly frequent, theoretically dependent on cardiac output, low systemic vascular resistance and hemolysis markers. In sickle cell disease (SCD), hyperfiltration is an extremely common phenomenon and occurred in young and early adult patients. Despite the fact that the glomerular hyperfiltration is known as the early manifestations of sickle cell nephropathy, its burden among adult sickle cell disease in sub-Saharan is poor studied. This study aimed to determine the prevalence and associated factors of hyperfiltration

**Methods:**

This was an analytical multicentric cross-sectional study involving stable adult sickle cell patients in Kinshasa, recruited between March and October 2023. Parameters of interest encompasses demographic, clinical, biological, echocardiographic and pulse wave measurement data. Hyperfiltration was defined using the CDK-EPI equation based on cystatin C; eGFR >130 for women and >140 ml/min/1.73m^2^ for men. We used multivariate logistic regression analysis to search determinants of glomerular hyperfiltration.

**Results:**

Two hundred and fourty six (246) patients with SCD were enrolled. The prevalence of hyperfiltration was 20.7%. In multiple logistic regression analysis, hyperfiltration status was independently associated with age (< 25 years) [3.57 (1.78-7.49); *p* = 0.027)], female sex [4.36 (2.55-5.62); *p* = 0.031), CRP (< 6 mg/l) [0.77 (0.61-0.97); *p* = 0.028)], central systolic pressure (< 100 mmHg) and central diastolic pressure (< 60 mmHg) [0.86(0.74-0.98), *p* = 0.028)], [(0.83 (0.71-0.98); *p* = 0.032)].

**Conclusion:**

One out of five SS adults exhibits hyperfiltration, which is associated with young age and female sex, whereas low CRP and blood pressure were negative risk factors.

## Introduction

The renal complications arising from sickle cell disease are now a major issue, as they are the cause of renal failure affecting approximately 12 to 21% of young patients [1] and 80% of old patients [2,3]. The particular renal hemodynamic changes in sickle cell disease are characterized in the initial phase by hyperfiltration [4], followed by sickle cell nephropathy, clinically marked by glomerular damage and significant proteinuria, which progresses insidiously toward CKD [3,5]. This glomerular hyperfiltration, which is the early phase, is similar to that of type 1 diabetic nephropathy. In diabetes, it also results from neuro-hormonal, vascular and even tubular factors causing an effective decrease in afferent arteriolar resistance and an increase in afferent resistance leading to an increase in GFR then to glomerulopathy and ultimately to chronic kidney disease [6]. Hyperfiltration is variably defined as a glomerular filtration rate (GFR) ≥ 140 mL/min/1.73 m^2^ [7] and appears to continue to progress into early adulthood [8]. Almost all adults aged between 18 and 30 also have an elevated creatinine-based eGFR ≥ 130 mL/min/1.73 m^2^ [9], but this declines from the age of 30 to 40. This hyperfiltration is very prevalent, ranging from 60 to 80% in children and 50 to 60% in young adults [1,4,10]. The increase in GFR is due to an unusually high renal plasma flow with a low, but sometimes normal, filtration fraction [11], combined with increased cardiac output [12] and low arterial pressure, suggesting low systemic vascular resistance in most sickle cell patients [13]. Hemolysis also promotes the pronounced action of hem oxygenase in response to oxidative stress, resulting in the production of carbon monoxide (CO), which has vasodilatory properties [14]. The release of Hb, endothelin-1 (ET-1) and xanthine oxidase (XO) by hemolysis leads to the trapping and increased consuming of NO. This promotes the release of vasoactive hormones, cell adhesion, vaso-occlusion and hence endothelial damage [15].

This glomerular hyperfiltration also underlies proximal tubular hyperfunction and creatinine hypersecretion, leading to low creatinine levels in sickle cell patients compared with controls, suggesting that renal dysfunction may be underestimated by equations used to estimate creatinine clearance. Hence the best method of assessing this hyperfiltration would be to measure GFR using substances such as inulin, iothalamate, iohexol, 51-Cr EDTA. Though this method is difficult and rarely used in clinical practice. Hence, we resorted to cystatin C, which is better than creatinine since it is freely filtered, not secreted and entirely catabolized in the proximal tubules, although it can also be influenced by factors such as diabetes mellitus [16], age and inflammation [17], and administration of corticosteroids [18]. To our knowledge, the burden of hyperfiltration using cystatin C based CKD-EPI equation as well as cardiovascular parameters associated with eGFR in sub-Saharan have not been studied so far.

The presented study aimed to determine the prevalence and associated factors of glomerular hyperfiltration using the cystatin C-based CKD-EPI equation in young adults with SS in Kinshasa.

## Materials and methods

### Patient population and methods

This was an analytical multicenter cross-sectional study encompassing stable adult sickle cell patients in Kinshasa (DR Congo), recruited from 11 centers (SS Anemia Medical Mixte Center, Makala General Reference Hospital, Bonkoko Medical Center, Akram Hospital, Messie Center, Elisabeth Clinic, Amza Foundation, Rezodrepa, Codek, Colombe, Lisungi) between March and October 2023. Ethics approval and consent to participate with national ethical laws n°: ESP/CE/143/2022.

**Figure 1.**
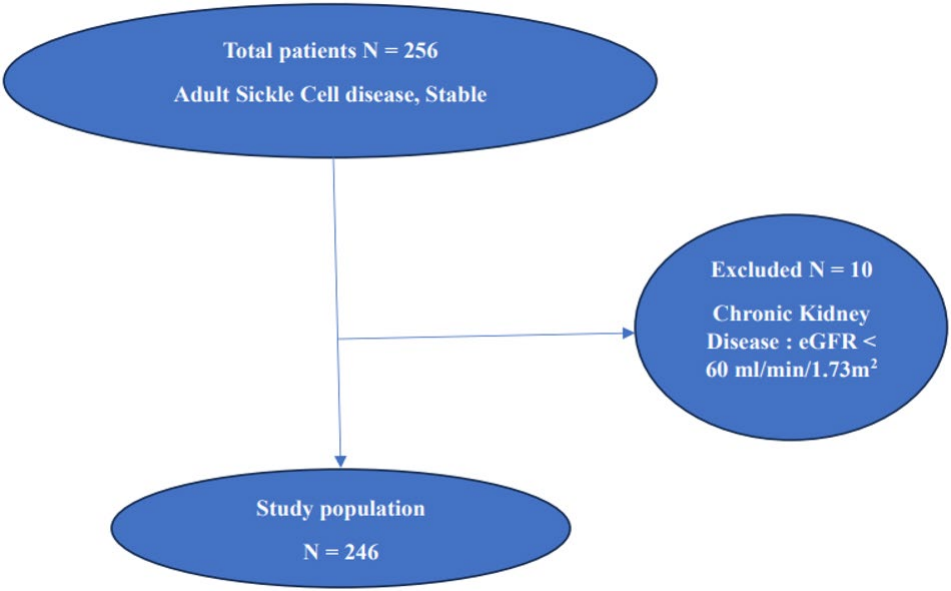
Flow chart of the study population.

All patients with stable sickle cell disease were included in this study, i.e., absence of vaso-occlusive crisis, infection, hospitalization for at least one month, acute chest syndrome (ACS), fever, pregnancy, urinary tract infection or use of aminoglycosides or non-steroidal anti-inflammatory drugs in the last month. On the other hand, those with a history of diabetes mellitus, viral hepatitis B or C, or HIV after biological examinations were not included. Those with an eGFR < 60 mL/min/1.73 m^2^ were excluded (Figure 1).

We collected clinical and laboratory values of interest at the time of the visit. We measured creatinine using an enzymatic technique and then cystatin C, CRP, ferritin and microalbuminuria by immuno-nephelometry using an I-Chroma 2 analyzer (Seoul, South Korea). Complete blood counts were performed by impedance analysis using a Zybio analyzer (Seoul, South Korea). LDH, AST, total and direct bilirubin were determined by kinetic method using a semi-automated system. Hemoglobin S were also quantified by cellulose acetate electrophoresis using the Gazelle machine (Hemex Health, Portland, USA).

Estimated GFR (eGFR) was calculated according to Chronic Kidney Disease - Epidemiology (CKD-EPI 2009) [19].

$$\begin{array}{c} {\text{GFR}}_{\text{cys}}=\text{ 133}\times \text{ min}{\left(\text{cys}/0.8,1\right)}^{-0.499}\times \text{ max}{\left(\text{cys}/0.8,1\right)}^{1.328}\\ \times \;0.996^{\text{Age}}\times \;0.932\left(\text{female}\right) \end{array}$$

$$\begin{array}{c} {\text{GFR}}_{\text{creat}}=\text{ 141 }\times \text{ min}{\left(\text{Scr}/k,\text{ 1}\right)}^{\alpha }\;\times \text{ max}{\left(\text{Scr}/k,\text{ 1}\right)}^{-1.209}\;\times \;0,993^{\text{Age}}\;\\ \times \text{ 1}.018\;\left(\text{if female}\right)\;\times \text{ 1}.159\;\left(\text{AF}\right) \end{array}$$

$$\begin{array}{c} {\text{GFR}}_{\text{mix}}=\text{ 135 }\times \text{ min}{\left(\text{Scr}/k,1\right)}^{\alpha }\;\times \text{ max}{\left(\text{Scr}/k,1\right)}^{-0.601}\\ \times \text{ min}{\left(\text{cys}/0.8,1\right)}^{-0.375}\;\times \text{ max}{\left(\text{cys}/0.8,1\right)}^{-0.711}\;\times \;0.995^{\text{Age}}\\ \times \;0.969\;\left(\text{female}\right)\;\times \text{ 1}.08\;\left(\text{AF}\right). \end{array}$$

eGFR was calculated according to European Kidney Function Consortium (EKFC) [20]:

$$\text{EKFC }-\text{ eGFR }=\text{ 1}07.3/{\left(\text{cys}/Q\right)}^{\alpha }\;\times \;\left[0.990^{\left(\text{Age}-40\right)}\text{if age }>40\text{ years}\right],$$

AF: Afro; Scr: serum creatinine; cys: cystatin C; α = −0.329[if female] or −0.411[if male]; κ = 0.7[if female] or 0.9[if male]; min and max indicate the minimum or maximum of SCr/κ or 1, respectively; Q values described in detail elsewhere.

Urine albumin to creatinine ratio (ACR), mg/g, was defined as albuminuria grade A1 (< 30 mg/g), albuminuria grade A2 (30 – 300 mg/g) and albuminuria grade A3 (> 300 mg/g) [21] using dipsticks automatically read by device (Cypress, Belgium). Urinalysis was completed with simple urine dipsticks.

Transthoracic Doppler echocardiography was performed by on a Mindray Z 60 (Shenzhen, China) ultrasound machine a single experienced cardiologist in accordance with guidelines [22]. In diastole, we measured the following parameters: left ventricular end-diastolic diameter (LVEDD), Left ventricular mass (LVM) [23]. Cardiac output (CO) was calculated and indexed (CI) to body surface area according to recommendations [23]. Maximum tricuspid regurgitation was recorded in multiple views and the highest velocity level was selected. High pulmonary systolic pressure was defined as peak tricuspid regurgitation velocity (TRIV) >2.3 m/s [24]. Systemic vascular resistance (SVR = 80 x mean arterial ­pressure/CO). Low SVR was < 700 dynes/cm^5^.

Carotid to femoral pulse wave velocity (PWV) using foot-pulse velocity measurement (Popmeter, Axelfil, France) enabled us to determine aortic stiffness [25].

The patient aged ≥ 18 years was considered an adult; central systolic pressure and central diastolic pressure were measured using a no-invasive method of central aortic pressure by a high-precision tonometer with two sensors whose data were analyzed by computer [26]. Chronic kidney disease was defined as eGFR < 60 mL/min per 1.73 m^2^ [27] and glomerular hyperfiltration as eGFR > 130 mL/min per 1.73 m^2^ for women and > 140 mL/min per 1.73 m^2^ for men [28]. Patients who received 15 to 20 mg/kg of hydroxyurea daily for 6 months namely formed the hydroxyurea group [29], while those who received no hydroxyurea or a low dose for a shorter period formed the no hydroxyurea group.

### Statistical analysis

The data were compiled in an Excel 2010 database, checked for consistency and then exported to Statistical Package for Social Sciences version 26 (SPSS for Windows version 26) for analysis. Descriptive statistics were presented as the mean (plus or minus standard deviation) for continuous variables with a normal distribution and as the median (IQR: Interquartile Range) for continuous data with a no-Gaussian distribution. The normality test (Kolmogorov-Smirnov or Shapiro-Wilk) was used to differentiate between normally and no-normally distributed quantitative variables. Absolute (n) and relative (%) frequencies were expressed for categorical variables. The student t-test, Mann-Whitney U-test and Pearson Chi-square or Fischer exact test were used to compare means, medians and proportions in the two groups, respectively. The linear regression test was applied to check the correlation between eGFR and the quantitative variables (Hb, PWV and LVM), and the Spearman coefficients (Rhô) of linear regression in simple analysis were calculated to assess the association between eGFR and these 3 variables. Logistic regression was used to search for the determinants of hyperfiltration in univariate and multivariate analysis, with calculation of the OR and their 95% confidence intervals.

## Results and discussion

Two hundred and fourty six (246) patients were included in the study (Figure 1), 91 (46.8%) were men. The mean age was 26.6 ± 8.1 years. The mean GFR of the study population was 110.9 ± 24.3 mL/min/1.73m^2^ and 136.1 ± 6.9 mL/min/1.73m^2^ for the hyperfiltration group.

**Figure 2.**
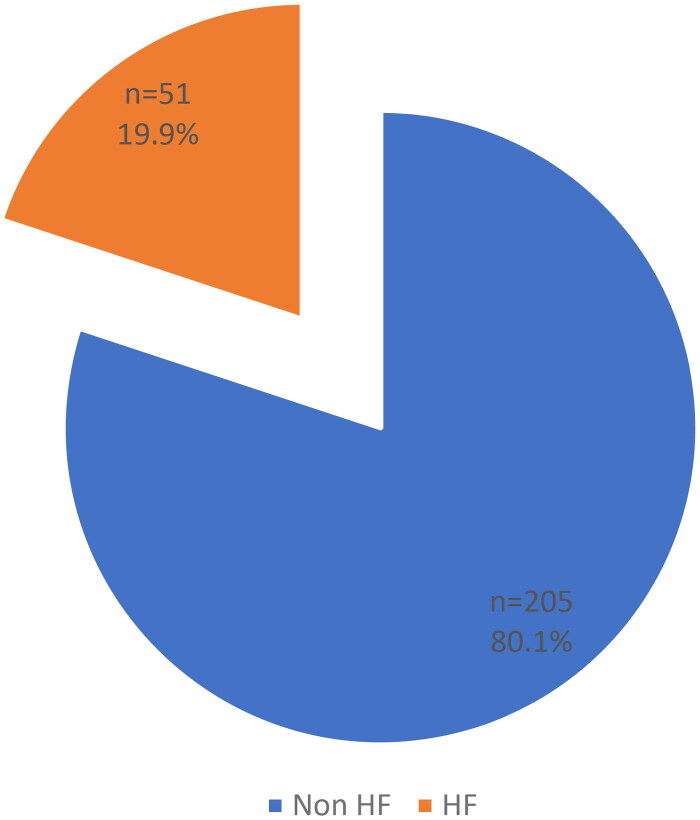
Prevalence of hyperfiltration in study population.

The mean Cystatin C values of 0.78 for the study population and 0.59 mg/dl for hyperfiltration group. Figure 2 illustrated the prevalence of hyperfiltration among all population studied. The prevalence of hyperfiltration assessed by the CKD-EPI equation based on cystatin C was 19.9%, i.e., 51 out of 256 patients in this series (Figure 2). According the CKD-EPI equation based on creatinine this prevalence increases to 42.3%; CKD-EPI equation combined with creatinine and cystatin C to 49.2%, whereas for the EKFC formula with cystatin, it is only 2%.

Table 1 presents the characteristics of patients studied according to the hydroxyurea status. The proportions of patients with folic acid, alcohol and HbF < 10 g/dl in group without hydroxyurea group were significantly higher than in hydroxyurea group. The proportion of family history SS and aseptic necrosis femoral were low in the hydroxyurea group compared to those without hydroxyurea group.

**Table 1.** General characteristics of patients studied according the hydroxyurea status.

Variables	No Hydroxyurea (*n* = 144)	Hydroxyurea (*n* = 102)	All (*n* = 246)	p
Age				0.414
<25 years	73(50.7)	54(52.9)	127(51.6)	
≥25 years	71(49.3)	48(47.1)	119(48.4)	
Sex				0.120
Female	91(63.2)	56(54.9)	147(59.8)	
Male	53(36.8)	46(45.1)	99(40.2)	
Profession				0.246
Student	64(44.4)	51(50.0)	115(46.7)	
Company employee	14(9.7)	15(14.7)	29(11.8)	
Civil servant	14(9.7)	6(5.9)	20(8.1)	
Unemployed	45(31.3)	27(26.5)	72(29.3)	
Housewife	7(4.9)	3(2.9)	10(4.1)	
Folic acid	128(89.5)	101(99.0)	229(93.5)	0.002
Surgery	34(23.8)	25(24.5)	59(24.1)	0.506
Family story of sickle cell	94(65.7)	57(55.9)	151(61.6)	0.031
Transfusion	132(92.3)	97(95.1)	229(93.5)	0.275
Skin ulcer	34(23.8)	28(27.5)	62(25.3)	0.306
Priapism	10(7.0)	10(9.8)	20(8.2)	0.287
Aseptic necrosis of femoral head	23(16.1)	24(23.5)	47(19.2)	0.045
Osteomyelitis	1(0.7)	1(1.0)	2(0.8)	0.660
Stroke	2(1.4)	4(3.9)	6(2.4)	0.197
Tobacco	15(10.5)	8(7.8)	23(9.4)	0.320
Alcohol	42(29.4)	19(18.6)	61(24.9)	0.038
NSAID	123(86.0)	90(88.2)	213(86.9)	0.379
CRP ≥ 6 mg/L	86(59.7)	68(66.7)	154(62.6)	0.058
HbF < 10	74(63.8)	38(52.1)	112(59.3)	0.034
Albuminuria				
Albuminuria grade A1	55(45.5)	38(46.3)	93(45.8)	0.507
Albuminuria grade A2	62(51.2)	37(45.1)	99(48.8)	0.294
Albuminuria grade A3	4(3.3)	7(8.5)	11(5.4)	0.167
Low SVR	12(21.8)	4(17.4)	16(20.5)	0.226
Nephromegaly	13(18.3)	8(27.6)	21(21.0)	0.220
Hepatomegaly	23(32.4)	9(31.0)	32(32.0)	0.546
Splenomegaly	3(4.2)	1(3.4)	4(4.0)	0.670

Data are expressed as mean ± standard deviation, absolute (n) and relative (in percent) frequencies. Abbreviations: ACEI or ARB II: ACE inhibitor or Angiotensin II receptor antagonist; CRP: C reactive protein; HbF: Fetal hemoglobin; Low SVR: Low Systemic Vascular Resistance; NSAID: No steroid anti-inflammatory; VOC: vaso-occlusive crisis.

Table 2 the mean age of patients with hyperfiltration was 22.3 ± 4.4 years, with 78% under the age of 25 years old. Women was significantly preponderant than men with a sex ratio of 5:1. 47% of patients on hydroxyurea exhibited hyperfiltration. Hyperfiltration patients were much more likely to have retinopathy [25 (12.9%) vs 13 (25.5%), *p* = 0.027], and aseptic necrosis of the femoral head [42 (21.6%) vs 5(9.8%), *p* = 0.038] than those without. The proportion of albuminuria grade A2 and albuminuria grade A3 was similar between the two groups (no hyperfiltration versus hyperfiltration).

**Table 2.** General characteristics of study population according to hyperfiltration.

Variables	No Hyperfiltration (*n* = 195)	Hyperfiltration (*n* = 51)	Overall (*n* = 246)	p
Age (years)	27.7 ± 8.6	22.3 ± 4.4	26.6 ± 8.1	<0.001
Age <25 years	87(44.6)	43(84.3)	147(59.8)	<0.001
Sex				<0.001
Female	104(53.3)	43(84.3)	147(59.8)	
Male	91(46.8)	8(15.7)	99(40.6)	
Folic acid	181(93.3)	48(94.1)	229(93.5)	0.564
Hydroxyurea	78(40.0)	24(47.1)	102(42.0)	0.226
Skin ulcer	49(25.3)	13(25.5)	62(25.3)	0.552
Priapism	18(8.8)	2(3.9)	20(7.8)	0.195
Retinopathy	25(12.9)	13(25.5)	38(15.5)	0.027
Aseptic necrosis of femoral head	42(21.6)	5(9.8)	49(19.2)	0.038
Stroke	5(2.6)	1(2.0)	6(2.4)	0.637
Tobacco	19(9.8)	4(7.8)	23(9.4)	0.522
Alcohol	48(24.7)	13(25.5)	61(25.1)	0.536
CRP ≥6 mg/L	129(66.2)	25(49.0)	154(62.6)	0.019
HbF ≥10%	61(39.9)	16(44.4)	77(40.7)	0.374
Low SVR	14(20.3)	2(13.3)	16(19.0)	0.417
Albuminuria				0,407
Albuminuria grade A1	73(45.1)	20(48.8)	93(45.7)	
Albuminuria grade A2	82(50.6)	17(41.5)	99(48.8)	
Albuminuria grade A3	7(4.3)	4(9.8)	11(6.2)	

Data are expressed as mean ± standard deviation, absolute (n) and relative (in percent) frequencies. Abbreviations: CRP: C reactive protein; HbF: hemoglobin Fetal; Low SVR: Low Systemic Vascular Resistance.

Table 3 as expected, variables such as cystatin C, creatinine and urea showed a significant difference. The other GOT and CRP were also significant [34.7(26.8-48.6) vs 30.6(27.1-34.1), *p* = 0.043] and [9.8(3.6-24.8) vs 5.5(2.5-10.2), *p* = 0.024], between the no hyperfiltration group and hyperfiltration group. But there was no statistically significant difference in HbF levels between the two groups [9.0(7.3 − 12.0) vs 9.0(8.0 − 12.8), *p* = 0.724]. One significant difference urine albumin to creatinine ratio was observed. Only 47.8% of patients with hyperfiltration had no albuminuria grade A1, compared with 41.5% with albuminuria grade A2 and 9.8% with albuminuria grade A3.

**Table 3.** Biological characteristics of the population studied according to hyperfiltration.

Variables	No Hyperfiltration (*n* = 195)	Hyperfiltration (*n* = 51)	Overall (*n* = 246)	p
Cystatin, mg/dL	0.83(0.73-0.96)	0.59(0.52-0.63)	0.78(0.65-0.92)	<0.001
eGFR_CKD-EPI	104.6 ± 23.0	136.1 ± 6.9	110.9 ± 24.3	<0.001
Creatinin, mg/dL	0.9(0.7-1.0)	0.7(0.6-0.8)	0.8(0.7-1.0)	0.021
Urea, mg/dL	5.2 ± 1.7	4.1 ± 1.3	4.9 ± 1.8	<0.001
Hb, g/dL	7.4 ± 1.8	7.6 ± 1.2	7.4 ± 1.7	0.355
Hct, %	23.1 ± 6.0	23.5 ± 3.9	23.2 ± 5.7	0.639
GR10^6^/mm^3^	2.7 ± 0.8	2.7 ± 0.5	2.7 ± 0.7	0.973
Reticulocytes, %	16.9 ± 2.2	16.3 ± 1.5	16.8 ± 2.1	0.053
WBCx10^3^/mm^3^	11.3(8.9-14.6)	10.5(8.0-13.4)	11.1(8.9-14.3)	0.129
Platelets /mm^3^	161.8 ± 22.3	159.9 ± 11.6	161.4 ± 20.6	0.540
ASAT, IU	34.7(26.8-48.6)	30.6(27.1-34.1)	33.2(26.9-46.2)	0.043
Total Bili, mg/dL	1.0(0.94-1.30)	0.99(0.90-1.10)	1.0(0.9-1.2)	0.221
Direct Bili, mg/dL	0.31(0.25-0.67)	0.3(0.24-0.41)	0.31(0.25-0.62)	0.176
Indirect Bili, mg/dL	0.68(0.53-0.77)	0.68(0.51-0.76)	0.68(0.53-0.77)	0.577
LDH, U/l	221.0(196.6-301.6)	217.3(190.4-241.1)	219.0(196.2-290.0)	0.314
Ferritin, ng/mL	292.0(92.9-845.4)	177.7(81.6-725.0)	264.1(88.8-833.4)	0.151
CRP, mg/L	9.8(3,6-24.8)	5.5(2.5-10.2)	9.1(3.5-21.1)	0.024
HbS, %	85.1 ± 14.6	81.3 ± 23.3	84.4 ± 16.6	0.224
HbF, %	9.0(7.3-12.0)	9.0(8.0-12.8)	9.0(8.0-12.0)	0.724
Urinary density	1.02 ± 0.004	1.02 ± 0.005	1.02 ± 0.004	0.989
Urinary pH	6.0 ± 0.4	6.0 ± 0.4	6.0 ± 0.4	0.940
ACR, mg/g	40.0(20.0-80.0)	30.0(10.0-50.0)	30.0(20.0-80.0)	0.267

Data are expressed as mean ± standard deviation, median (interquartile range), absolute (n) and relative (in percent) frequencies. Abbreviations: ACR: albuminuria to creatinuria ratio; ASAT: Aspartate aminotransferase; Bili: Bilirubin; CRP: C-reactive protein, GFR-CKD: Glomerular filtration rate-Chronic Kidney Disease; Hb: Hemoglobin; HbF: Hemoglobin fetal; HbS: Hemoglobin S; Hct: Hematocrit; LDH: Lactico dehydrogenase, MCCH: Mean corpuscular concentration hemoglobin; MCH: Mean corpuscular hemoglobin; MCV: Mean corpuscular volume; pH: Hydrogen potential; RBC: Red blood cells; WBC: White blood cells.

In the Table 4, the patients with low CSP and CDP had significantly more hyperfiltration than those with high pressures (*p* < 0.05). They also had a lower PWV than those without hyperfiltration, but this statistical difference was not significant. The majority of patients had a normal LVEF and there was no statistically significant difference between the both groups. The mean TRIV was 1.71 ± 0.34 m/sec, and there was no statistically significant difference between the hyperfiltration group and without. Only one patient with a TRIV ≥ 2.3 m/sec had hyperfiltration. Cardiac output was significant slightly lower in patients with hyperfiltration (6.8 ± 2.3 vs 6.6 ± 1.4; *p* = 0.008). SVR was low in the hyperfiltration group but there was no significant difference with the no hyperfiltration group (929.7 ± 191.3 vs 953.1 ± 366.0, *p* = 0.531).

**Table 4.** Hemodynamic and echocardiographic characteristics.

Variables	No Hyperfiltration (*n* = 195)	Hyperfiltration (*n* = 51)	Overall (*n* = 246)	p
PWV, m/s	5.1(4,5-6,1)	4.7(4.0-5.2)	5.0(4.4-5.9)	0.157
TT,	105.3 ± 22.9	114.8 ± 21.6	107.6 ± 22.8	0.106
CSP, mmHg	98.9 ± 12.9	92.2 ± 8.0	97.3 ± 12.3	0.031
CDP, mmHg	62.9 ± 10.9	57.0 ± 8.9	61.5 ± 10.7	0.031
PP, mmHg	35.9 ± 8.0	35.2 ± 9.0	35.7 ± 8.2	0.730
HR, bpm	82.6 ± 15.6	82.9 ± 7.7	82.7 ± 14.2	0.945
ABI,	1.1 ± 0.06	1.1 ± 0.06	1.1 ± 0.07	0.258
IVS, mm	8.8 ± 1.7	8.6 ± 1.3	8.8 ± 1.6	0.712
PP, mm	9.2 ± 1.7	8.8 ± 1.5	9.1 ± 1.7	0.379
LVEF, %	67.4 ± 9.7	68.4 ± 10.0	67.6 ± 9.7	0.684
SF, %	38.4 ± 8.0	39.0 ± 7.7	38.5 ± 7.9	0.768
LVEDD, mm	50.7 ± 5.4	49.9 ± 4.8	50.5 ± 5.3	0.555
RVEDD, mm	28.3 ± 4.7	26.1 ± 5.0	27.9 ± 4.8	0.076
LVM, g/m^2^	166.1 ± 43.6	152.8 ± 35.6	163.8 ± 42.4	0.215
LVMi, g/m^2^	110.0 ± 27.4	104.6 ± 20.6	109.0 ± 26.3	0.422
ICV, mm	13.0 ± 3.2	12.6 ± 2.6	12.9 ± 3.1	0.636
E/A	1.4 ± 0.3	1.6 ± 0.3	1.5 ± 0.3	0.063
E/E’	7.8 ± 1.9	7.3 ± 1.6	7.7 ± 1.8	0.310
DT, sec	0.15 ± 0.05	0.15 ± 0.04	0.15 ± 0.05	0.922
TRIV (m/sec)	1.71 ± 0.33	1.70 ± 0.38	1.71 ± 0.34	0.933
TRIV cat				0.463
<2.3	97(93.3)	27(96.4)	124(93.9)	
≥2.3	7(6.7)	1(3.6)	8(6.1)	
RA Area, cm^2^	14.4 ± 3.2	12.9 ± 2.2	14.1 ± 3.1	0.057
LA Area, cm^2^	18.2 ± 3.7	17.3 ± 3.3	18.0 ± 3.6	0.329
TAPSE	2.7 ± 0.4	2.7 ± 0.04	2.7 ± 0.4	0.970
CI	4.6 ± 1.6	4.6 ± 1.0	4.6 ± 1.5	0.902
Cardiac output, l/’	6.8 ± 2.3	6.6 ± 1.4	6.8 ± 2.2	0.008
SVR, dyn s cm^-5^	953.1 ± 366.0	929.7 ± 191.3	949.0 ± 341.2	0.531

Data are expressed as mean ± standard deviation, median (interquartile range), absolute (n) and relative (in percent) frequencies. Abbreviations: ABI: Ankle brachial index; CDP: Central diastolic pressure; CI: Cardiac index; CSP: Central systolic pressure; DT: Deceleration time; E/A: The mean of the septal and lateral E waves in tissue Doppler at the level of the mitral annulus; IVC: Inferior vena cava; IVS: Interventricular septum; LA area: Left atrial area; LVEDD: Left ventricular end-diastolic diameter; LVEF: Left ventricular ejection fraction; LVM: Left ventricular mass; LVMi: Indexed left ventricular mass; PP: Pulse pressure; PP: Posterior wall; PWV: Pulse wave velocity; RA area: Right atrial area; RVEDD: Right ventricular end-diastolic diameter; TAPSE: Tricuspid annular plane systolic excursion;; TRIV: Tricuspid regurgitant jet velocity; TT: Transit time; SF: Shortening fraction; SVR: Systemic vascular resistance.

**Figure 3.**
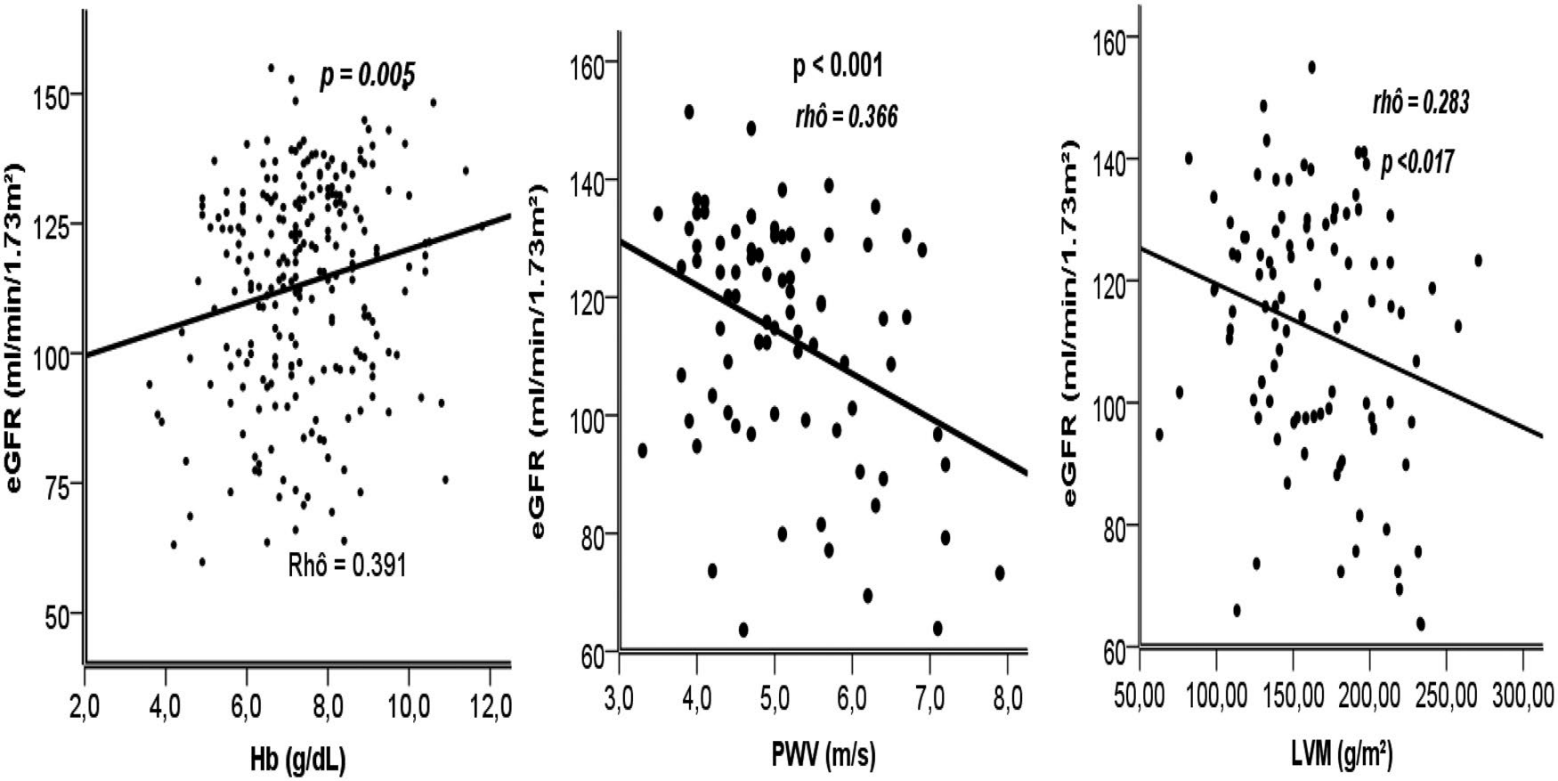
Correlation with estimated glomerular filtration rate.

eGFR CKD-EPI was significantly associated with Hb, PWV and LVM. High eGFR values were associated with high Hb (rho = 0.371, *p* < 0.001) and low eGFR values with high PWV and LV mass (rho = 0.366, *p* < 0.001), (rho = 0.283, *p* < 0.017) (Figure 3).

Using multiple logistic regression analysis (Table 5), we found that hyperfiltration status was independently associated with age (< 25 years) [3.57 (1.78-7.49); *p* = 0.027)], female sex [4.36 (2.55-5.62); *p* = 0.031), CRP (< 6 mg/l) [0.77 (0.61-0.97); *p* = 0.028)], CSP (< 100 mmHg) and CDP (< 60 mmHg) [0.86(0.74-0.98), *p* = 0.028)], [(0.83 (0.71-0.98); *p* = 0.032)], suggesting low vessel resistance.

**Table 5.** Factors associated with hyperfiltration of study population.

Variables	Univariate analysis		Multivariate analysis			
			Model 1		Model 2	
	P	OR (CI 95%)	p	aOR (CI 95%)	p	aOR (CI 95%)
Age						
≥25 years				1		1
<25 years	<0.001	4.56(2,21-9,38)	0.027	3.57(1.78-7.49)	0.029	5.04(2.68-7.18)
Sex						
Male		1		1		1
Female	<0.001	4.73(2.12-10.57)	0.031	4.36(2.55-5.62)	0.037	4.58(2.26-7.24)
ASAT*	0.029	0.98(0.96-0.99)	0.122	1.04(0.99-1.08)	0.082	1.04(0.99-1.08)
CRP*	0.036	0.98(0.96-0.99)	0.028	0.77(0.61-0.97)	0.020	0.77(0.62-0.96)
CSP*	0.036	0.94(0.89-0.99)	–	–	0.028	0.86(0.74-0.98)
CDP*	0.032	0.94(0.88-0.99)	0.032	0.83(0.71-0.98)	–	–
Cardiac output*	0.043	0.72(0.57-0.96)	0.090	0.42(0.16-1.15)	0.067	0.42(0.16-1.06)

ASAT: Aspartate aminotransferase; CDP: Central diastolic pressure, CRP: C-reactive protein; CSP: Central systolic pressure.

## Discussion

Our results show that the mean age was 26.6 ± 8.1 years in the sickle cell population and 22.3 ± 4.4 years in those with hyperfiltration, with 78% under 25 years of age. Depend on the equation used, the prevalence of hyperfiltration varies from 19.9% (by CKD-EPI cystatin C), 42.3% (by CKD-EPI creatinine), 49.2% (CKD-EPI combined creatinine, cystatin C) and 2% (by EKFC cystatin C). The determinants independently associated with hyperfiltration found in this study population were age under 25, female gender, CRP and central systolic and diastolic pressures.

Compared with Haymann’s 51% with MDRD equation and Marouf’s 30.5% and 44% with MDRD equation and Cockroft-Gault equation respectively [1,28], our prevalence of hyperfiltration appears lower and can be explained by the use of different equations and biomarkers, although the criteria for hyperfiltration remain the same. The use of creatinine with body weight in the Cockroft-Gault formulae is highly biased because of the muscle wasting, hypersecretion and hyperfiltration seen in sickle cell patients [1]. We also included only stable sickle cell patients and excluded all those who had presented with VOC, use of aminoglycosides or non-steroidal anti-inflammatory drugs or were transfused within less than one month. A much higher prevalence was also found in some studies when only homozygous HbSS sickle cell patients were included, excluding cases of ß thalassemia [9]. The prevalence of this series is higher when compared with the 10% yielded by Marouf’s using an equation based on cystatin C. GFR estimated on the basis of cystatin C correlated more closely than that measured by nuclear methods in several series in sickle cell patients [30,31]; although this study using GFR measured with 51-Cr EDTA showed a much higher prevalence of hyperfiltration at 81% [32]. At present, the cystatin-based CKD-EPI equation is used to estimate GFR in adults with sickle cell disease, as it is less biased and more accurate than the combined equation with creatinine and cystatin C when compared with GFR measured using iohexol [33]. Kim et al. revealed that the transition from the CKD-EPI equations to the EKFC overestimated the prevalence of chronic renal failure, and therefore reduced that of hyperfiltration, although the difference was still greater in our study [34].

This difference in the estimation of GFR shows the lack of precision and the need for its measurement. This difference has also been observed by Ndour et al. in Senegal, who found a prevalence of 7% for JSCSS, 33.9% for CKD-EPI race, 78.5% for MDRD and 66.1% for CKD-EPI without racial factors [35].

The expected process in the occurrence of hyperfiltration is the increase in cardiac output and the decrease in systemic vascular resistance as reported in several studies [32] but in our series the cardiac output was rather low compared with the group without hyperfiltration even though vascular resistance was low and found in 14%. Systolic and diastolic blood pressures are often normal in sickle cell disease [36]. Poor urine concentration or hyposthenuria accompanied by excessive sodium loss may also justify low blood pressure [37]. This low SVR value is combined with low central systolic and diastolic pressures, which appear to be protective factors in this multivariate logistic regression analysis. Normal or low arterial pressure in sickle cell nephropathy, in contrast to diabetic or obesity-related nephropathy, may justify the impact of low filtration fraction as another factor influencing glomerular hyperfiltration [38].

Although we found no statistical difference between HbF levels in the hyperfiltration and no-hyperfiltration groups, patients without hydroxyurea, which inhibits ribonucleotide reductase, thereby decreasing hemoglobin S and increasing hemoglobin F [39] still had lower HbF levels than patients with hydroxyurea. This can also be explained by the relatively low doses of hydroxyurea that these patients receive, since glomerular filtration rate is not often used as a basis for prescribing, whereas most of them have glomerular hyperfiltration even at an early stage [40].

Some studies, such as HUSTLE, in contradiction with others, have demonstrated the impact of hydroxyurea on the fall in GFR and probably on hyperfiltration [41], although there was no clear difference in this series between patients with hyperfiltration with or without hydroxyurea. Hyperfiltration often appears at a young age around 7 to 13 years and persists into adulthood in the 2 decades of life [42] with a prevalence of 25% [43] and regression which is most often accompanied by renal damage often expressed by persistent proteinuria [44].

The predominance of the female sex in our study may be justified by the fact that renal function declines more rapidly in men than in women after about 20 years after birth [45], whereas in the definition of hyperfiltration, the threshold in women of 130 mL/min/1.73m^2^ is even lower than in men. This was confirmed by a study in sickle cell mice, in which hyperfiltration appeared earlier, with a rapid fall in glomerular filtration rate in males, in contrast to females, who had a very slow fall [46] and a dissimilar pattern of ET-1 and glomerular porosity [47]. Given that our series only includes adults, it is possible that the found that their eGFR was probably already decreasing due to early hyperfiltration that had not been evaluated.

Concerning albuminuria, some studies showed that 9 out of 10 sickle cell patients aged at least 20 years had an eGFR >140 mL/min/1.73 m^2^ [8,48]. Since hyperfiltration always precedes the onset of albuminuria, which is a factor in progression to chronic renal failure, practitioners should monitor its presence and especially its absence at the beginning of adult age, which is a sign of an advanced process in patients with sickle cell disease [44].

In our study, there was an association between CRP and hyperfiltration; as CRP fell, glomerular filtration rate fell. Low CRP appears have negative risk against hyperfiltration. Inflammation is part of the pathophysiology of glomerular hyperfiltration [49]; it has been shown that the use of non-steroidal anti-inflammatory drugs (which inhibit the production of prostaglandins) is accompanied by a fall in GFR [50]. This is consistent with studies showing that inflammation is a factor in hyperfiltration. In contrast, there is an inverse association for hemoglobin; as hemoglobin increases, glomerular filtration rate also increases. This is incompatible with several studies in which hemolysis, which leads to anemia, contributed to the onset of hyperfiltration. Is this the effect of hydroxyurea, which normally reduces hemolysis and inflammation and increases hemoglobin levels, since almost half the patients with hyperfiltration were receiving it? Or is it a reflection of early hyperfiltration with a subsequent fall in GFR until normalization. Nitric oxide, provided by hydroxyurea, promotes vasodilatation with an increase in renal plasma flow and may also underlie hyperfiltration [51].

An inverse association has also been observed for LVM and GFR. An increase in LVM was accompanied by a decrease in GFR. This can be explained by the fact that the sickle cell heart compensates for the anemia by increasing cardiac output and decreasing vascular resistance, thereby increasing left ventricular pressures and parietal stress, leading to left ventricular hypertrophy (LVH) [52]. This LVH provides the basis for myocardial fibrosis, which causes a lack of cardiac stretch and contractility, leading to diastolic dysfunction [53].

Pulse wave velocity, a reflection of aortic stiffness, is a predictive and prognostic factor in cardiovascular and chronic renal morbidity and mortality [54]. In sickle cell disease, its elevation is often associated with endothelial dysfunction and hemolysis [55,56]. In our study, it was accompanied by a fall in GFR. This negative association can be explained either by an increase in blood viscosity or by a normal GFR, which is probably linked to normalization of early hyperfiltration that was not captured, thus showing the evolution of advanced chronic kidney disease.

In conclusion, despite possible methodological limitations, this present study emphasizes that hyperfiltration are highly prevalent in SCD, particularly among women aged under 25 years, but low CRP level and arterial pressure are associated with low risk for hyperfiltration. This disparity in prevalence in the same group of patients according to the different equations for assessing glomerular filtration rate shows us the need to measure this glomerular filtration rate, which will serve as a gold standard in order to find the best equation to use in sickle cell disease.

The main limitation of our study is that we did not measure glomerular filtration rate (GFR) in patients with sickle cell disease. The lack of use of certain assays to detect hemolytic and oxidative stress components such as haptoglobin. We also quantified HbF and HbS by cellulose acetate electrophoresis, which is not a reference method. This is the first study in DR Congo to assess glomerular filtration rate with cystatin C and our results are also strengthened by the availability of albumin/creatinine ratio values and pulse wave measurements. These are recognized and valuable screening tools for renal dysfunction by the published guidelines on sickle cell disease [57].

Longitudinal studies with GFR measurement and repeated quantification of proteinuria should also be carried out to support these hypotheses.

## Data Availability

The data can be made available to anyone who wishes to write to us at our email address.
